# The Enterotoxin Gene Profiles and Enterotoxin Production of *Staphylococcus aureus* Strains Isolated from Artisanal Cheeses in Belgium

**DOI:** 10.3390/foods12214019

**Published:** 2023-11-03

**Authors:** Raphaëlle Minutillo, Barbara Pirard, Abdelhak Fatihi, Marina Cavaiuolo, Donatien Lefebvre, Amaury Gérard, Bernard Taminiau, Yacine Nia, Jacques-Antoine Hennekinne, Georges Daube, Antoine Clinquart

**Affiliations:** 1 Department of Food Sciences, Fundamental and Applied Research for Animals & Health (FARAH), Faculty of Veterinary Medecine, Avenue de Cureghem 10, 4000 Liege, Belgiumbabs.pirard@gmail.com (B.P.); bernard.taminiau@uliege.be (B.T.); georges.daube@uliege.be (G.D.); 2 Laboratory for Food Safety, French Agency for Food, Environmental and Occupational Health & Safety (ANSES), Université Paris-Est, 94700 Maisons-Alfort, France; abdelhak.fatihi@anses.fr (A.F.); marina.cavaiuolo@anses.fr (M.C.); donatien.lefebvre@catalent.com (D.L.); yacine.nia@anses.fr (Y.N.); jacques-antoine.hennekinne@anses.fr (J.-A.H.); 3 Brewing and Food Science Unit, LABIRIS, Avenue Emile Gryzon 1, 1070 Anderlecht, Belgium; agerard@spfb.brussels

**Keywords:** cheeses, *Staphylococcus aureus*, staphylococcal enterotoxins, toxin detection, *egc*, food poisoning, Belgium

## Abstract

A *Staphyloccoccus aureus* is one of the leading causes of food poisoning outbreaks (FPOs) worldwide. Staphylococcal food poisoning (SFP) is induced by the ingestion of food containing sufficient levels of staphylococcal enterotoxins (SEs). Currently, 33 SEs and SE-like toxins (SEls) have been described in the literature, but only five named “classical” enterotoxins are commonly investigated in FPOs due to lack of specific routine analytical techniques. The aims of this study were to (i) establish the genetic profile of strains in a variety of artisanal cheeses (*n* = 30) in Belgium, (ii) analyze the expression of the SE(l)s by these strains and (iii) compare the output derived from the different analytical tools. Forty-nine isolates of *S. aureus* were isolated from ten Belgian artisanal cheeses and were analyzed via microbiological, immunological, liquid chromatography mass spectrometry, molecular typing and genetic methods. The results indicated that classical SEs were not the dominant SEs in the Belgian artisanal cheeses that were analyzed in this study, and that all *S. aureus* isolates harbored at least one gene encoding a new SE(l). Among the new SE(l)s genes found, some of them code for enterotoxins with demonstrated emetic activity and *ecg*-enterotoxins. It is worth noting that the involvement of some of these new SEs has been demonstrated in SFP outbreaks. Thus, this study highlighted the importance of the development of specific techniques for the proper investigation of SFP outbreaks.

## 1. Introduction

*Staphylococcus aureus* (*S. aureus*) is a pathogen that plays a role in many human illnesses [1] and one of the leading causes of food poisoning outbreaks (FPOs). *S. aureus* coagulase-positive strains (CPS) are well known as producing staphylococcal enterotoxins (SEs) in foods. The SEs are superantigens (powerful non-specific T-cell stimulators) that can also exhibit an emetic activity [2]. Milk and dairy products are among the most common foods associated with staphylococcal food poisoning (SFP) outbreaks [3,4]. This is true in Europe [3] but also in other continents, for example, in Asia [5]. SFP patients commonly exhibit a rapid onset of symptoms associated with acute gastroenteritis, including nausea, emesis, abdominal cramps and diarrhea [6]. SFP is mostly self-limiting and does not require intensive medical care in healthy or non-immuno-compromised individuals [2,7]. Notably, *S. aureus* is one of the main causative agents of mastitis in dairy ruminants, and thus, such infections may represent a potential source of this pathogen in dairy products [8]. Indeed, several studies have reported high prevalences of CPS [9,10,11,12,13], especially *S. aureus* [10,11], in raw milk and raw-milk cheeses. In addition, dairy products can be contaminated by *S. aureus* during processing due to human carriers and poor hygiene practices [14]. As this situation represents a potential risk for consumers, it may be necessary to control these pathogens during cheese manufacturing processes [15]. However, as these processes vary between different varieties of cheese, the prevalence and profile of *S. aureus* may also differ. For example, it has been shown that certain typical cheese making processes appear to inhibit the development of pathogenic microorganisms such as coagulase-positive *Staphylococcus* (CPS), *Salmonella*. spp. and *Listeria monocytogenes* [16,17].

SFP is induced by the ingestion of food containing a sufficient level of SEs. The typical SE doses that lead to food poisoning symptoms are very low, ranging from 20 to 100 ng [5], but they also vary depending on the toxin implicated [18]. The presence of approximately 10^5^ to 10^6^ CFU/g of CPS in food should induce the production of SEs, making the food unfit for consumption [19,20,21,22]. As a consequence, the Commission Regulation (EC) No. 2073/2005 on microbiological criteria for foodstuffs requires testing for the presence of SEs in unpasteurized cheese batches when the number of CPS exceeds 10^5^ CFU/g. However, the absence or low levels of CPS cannot exclude the presence of pre-formed SEs in food, as reported in several SFP outbreaks [23]. Indeed, after SE production, CPS counts can fall during the ripening process [24,25]. Moreover, unlike CPS, pre-formed SEs are relatively thermostable and can persist after standard thermal treatments employed in food manufacturing processes [5,26]. As a consequence, the conclusive confirmation of SFP outbreaks must rely on the detection of the toxins themselves in the consumed food and not on the CPS count and molecular typing only [19].

Different molecular typing methods can be used to compare *S. aureus* strains in outbreak investigations, including multiple-locus variable-number tandem repeat analysis (MLVA), multilocus sequence typing (MLST), spa-typing and pulsed-field gel electrophoresis (PFGE) [27,28,29].

The characterization of *S. aureus* food isolates can be performed using polymerase chain reaction (PCR) assays targeting 11 genes (*sea*, *seb*, *sec*, *sed*, *see*, *seg*, *sei*, *sep*, *sej*, *ser* and *seh*). These PCRs can be implemented as four triplex PCR assays, according to methods defined by the European Union Reference Laboratory for coagulase-positive staphylococci (EU-RL CPS) [30]. PCR has limitations: (i) it may not provide any information on the nucleotide variability; (ii) false positives can be obtained due to the amplification of pseudogenes and (iii) false negatives can be obtained due to mutations in the primer binding sites [31]. These limitations can be overcome using whole genome sequencing (WGS) [31]. However, molecular typing methods have two major limitations: (i) they require the isolation of staphylococcal isolates from the food matrix, and (ii) they are only based on DNA analysis and do not provide any information on the presence of SEs themselves in the food [32]. 

Currently, 33 SEs and SE-like toxins (SEls), i.e., those that have not been confirmed to have an emetic effect, have been described in the literature. Those designated as SEA to SEE are classified as “classical SEs”. These classic SEs all exhibit emetic activity and are the most commonly identified toxins (>75%) in SFP outbreaks worldwide [2,33,34]. Nevertheless, throughout the world, several *S. aureus* isolates derived from SFP outbreaks were shown to be negative for SEA to SEE toxins using routine techniques. Further extensive genetic analysis revealed that these isolates harbored “new or non-classical SE(l)” genes, indicating that new or non-classic SEs or SEls could also be the causative agents of SFP outbreaks [25,35,36,37]. For example, in two different food-borne outbreaks involving raw goat milk, only strains harboring *seg* and *sei*, and other genes encoded by the enterotoxin gene cluster (*egc*), were detected [24]. These new or non-classic SEs or SEls include SEG to SElZ, SEl26, SEl27, SEl28, SEl29, SEl30, SEl31, SEl32, SEl33 and the toxic shock syndrome toxin (TSST1), formerly referred to as SEF. Currently, there are several non-classic SEs that have either been proven to exhibit emetic activity or are suspected to be potential agents of food poisoning; these include SEA to SEE and SEG, SEH, SEI, SEK, SEL, SEM, SEN, SEO, SEP, SEQ, SER, SES, SET and SEY [32,38,39,40].

SE-encoding genes (*se*) are mostly located on mobile genetic elements such as plasmids, prophages or pathogenicity islands [1,41,42]. Among the genes coding for non-classic enterotoxins, six genes (*seg*, *sei*, *sem*, *sen*, *seo* and *selu*) are located on the *egc* cluster, which is part of the *S. aureus* genomic island *vSaβ* [43]. The genomic analysis of the strain allowed the identification of the origin (food poisoning outbreak, human carrier, animal carrier) and the type of contamination and can be used to predict the production of SEs [44]. The occurrence of SE(l) genes, and specifically *egc* genes, in *S. aureus* isolated from food is highly variable, which may be mainly related to the geographical and/or food type origin of isolates and to the methods used for their detection [4,12,45].

At this time, the diagnosis of SFP outbreaks is performed by the direct detection of classic SEs in the food matrix using immunological methods. Several methods are commonly used alone or in association to detect the presence of SEs in food: (1) bioassays and (2) immunological tools such as VIDAS SET2 and enzyme-linked immunosorbent assay (ELISA). At present, only five out of 33 known staphylococcal enterotoxins can be detected using commercially available kits [46,47]. However, the production of specific immunological tools for each SE can be difficult, as it requires developing specific antibodies for each SE [48]. Recently, e.g., a sandwich ELISA using mouse monoclonal antibodies has been developed to detect new SEs (SEG and SEI) [39]. Mass spectrometry-based methods have emerged as a promising technique, despite some limitations such as interference from complex matrices, high-cost analysis, low throughput, and the dependence on highly qualified operators. For instance, Lefebvre et al. [32] implemented an LC-MS method facilitating the detection of SEs in dairy food products, with a limit of quantification lower than 0.1 ng/g (in milk) and naturally contaminated samples of SFP outbreaks. Moreover, the same study showed that at least 24 SEs can be produced through a brain–heart infusion (BHI) by *S. aureus* strains, whereafter those SEs can be identified and quantified using the LC-MS method. 

Usually, studies associated with the genetic characterization of *S. aureus* strains and their potential to produce SEs are limited to one or two toxins, and in the majority of cases, such studies focus on classic SEs. Moreover, there are few studies that investigate the *S. aureus* strains present in foods outside of SFP outbreaks that involve the targeting of all SE types [19]. In this study, we analyzed Belgian cheese from 32 factories in order to (1) isolate and characterize *S. aureus* strains from Belgian artisanal cheeses at the beginning and at the end of their shelf life (D0 and DE, respectively) in three main types of cheese: unripened acid-curd, mold and smear-ripened soft and semi-hard cheeses in real cheese manufacturing conditions and (2) assess the capacity of these strains to produce SE(l)s, especially new SE(l)s, and particularly those encoded in the *egc-*cluster. 

## 2. Materials and Methods

### 2.1. Sampling

Thirty-two varieties of cheese were selected in order to be representative of the diversity of Belgian artisanal cheeses. Cheeses were produced between July 2018 and March 2019 and came from farms covering the entire Belgian territory. Samples analyzed have been collected as previously described by Gérard et al. [16]. They were classified based on three categories: (a) fresh cheeses (FC), i.e., unripened acid-curd cheeses (37.5%), (b) soft cheeses (SC), taking into account both mold-ripened soft unpressed cheeses and smear-ripened soft unpressed cheeses (typically red-crust cheese washed during ripening with water, brine or smear) (25%), and (c) semi-hard cheese (SHC), involving curd-pressed cheeses (37.5%). Two cheese varieties (22 and 30) were lost due to technical issues, and no data will be presented for these varieties. Therefore, 30 cheese varieties were finally considered for the study. Among these, some were made from either pasteurized milk (25%) or raw milk (75%) from different species (cow (78.1%), goat (12.5%) and ewe (9.4%)). Considered varieties were analyzed at two timepoints: at the end of production, i.e., the first day of their conservation period (D0), namely, at the end of ripening, or directly after draining for the ripened and fresh cheeses, respectively, and at the expiry date (DE), after a storage at 7 ± 1 °C throughout all shelf-life, which was dependent on the recommendation provided by respective producers. The complete information on the type of cheese, the type of milk used and the respective shelf-life in all cheeses are detailed in Table S2 in the Supplementary Materials.

### 2.2. Isolates and S. aureus Identification

Each selected cheese variety was named arbitrarily from 1 to 32 and was sampled in triplicate from the same production batch. Replicates were designated A, B and C. For staphylococcal detection on samples, stock suspensions (SSs) were constituted for each of the 30 cheeses at D0 and DE. All these suspensions were prepared as previously detailed in Gérard et al. [16], according to standard NF EN ISO 6887-5 (2020) [49]. Briefly, for each sample, a “rind-core” mixture was made using 25 g and a 10-fold dilution in 2% trisodium citrate dihydrate buffer. Homogenization was performed using Stomacher 400 (Seward, Worthing, UK). Suspensions were then stored at −20 °C until use, then thawed at 4 °C for one hour.

#### Stock Suspension Culture

Cheese batches that were positive for the presence of *S. aureus* were first identified by culture. Isolations were performed using 100 μL from each SS to inoculate Baird Parker medium supplemented with rabbit plasma fibrinogen (BP-RPF), followed by incubation at 37 °C for 24–48 h. This protocol was adapted from the NF EN ISO 6888–2:1999 method [50] and facilitated the distinguishing between coagulase-positive (CPS) and -negative (CNS) *Staphylococcus*. A cheese batch was considered positive for the presence of *S. aureus* when at least one of the three samples gave a positive SS culture on BP-RPF agar.

Staphylococcal isolates were then selected. Due to a strong presence of CNS on some plates, CPSs were difficult to pull out. We therefore picked a maximum of two different colonies per triplicate named, respectively, 1 or 2 on a non-selective medium, i.e., Columbia Agar containing 5% of sheep blood (Thermo Fisher Diagnostics B.V.B.A., Merelbeke, Belgium). The isolates were stored at −80 °C in 500 µL of BHI broth with glycerol 20%. Isolates were tested for the presence of catalase and Gram-stained. At the end of this step, catalase-negative or Gram-negative isolates were discarded. To conclude selection, identification was performed through amplicon sequencing in order to select only *S. aureus* strains. After DNA extraction (see below point Section 2.3.1), 16s rRNA genes of conserved isolates were amplified via classic PCR using Diamond *Taq*^®^ DNA Polymerase (Eurogenetec, Liège, Belgium) and 5′-GAGTTTGATCMTGGCTCAG-3′ and 5′-TACGGTTACCTTGTTACGAC-3′ as forward and reverse primers, respectively. Then, sequencing was implemented using GIGA Genomics platform (Liège, Belgium). Bacterial species were later confirmed using the online alignment tool BLAST^®^.

### 2.3. Quantification of S. aureus in Positive Cheese Samples

#### 2.3.1. DNA Extraction

The same kit and protocol were used for DNA extraction from positive cheese samples, as well as from aliquots from artificially inoculated cheese suspensions intended for quantification standard. Both extraction handlings were performed at two different times in order to avoid DNA contamination of samples. Total DNA was extracted from 1 mL of each positive cheese SS, and from 2 mL of each artificially inoculated standard cheese SS. The DNeasy Blood & Tissue kit (Quiagen Benelux B.V., Hulsterweg, The Netherlands) was used, following manufacturer’s recommendations. Cell wall lysis was performed by adding to cell extracts XX Lysostaphin and incubating for 2 h at 37 °C. A negative control with nuclease-free water was included. Purified DNA was eluted in 100 µL of buffer AE (molecular water), and its concentration was assessed using a NanoDropTM 2000 spectrophotometer (Isogen Life Science B.V., Sint-Pieters-Leeuw, Belgium). Extracts were then stored at −20 °C until use.

#### 2.3.2. Quantitative PCR Amplification

Quantitative PCR (qPCR) was performed using Takyon (NoRoxProbeUNG) master mix with carry-over prevention (Eurogentec, Liège, Belgium), targeting the *nuc* gene of *S. aureus*. TaqMan probe sequence was 5′-TGAAGTCGAGTTTGACAAAGGT-3′ labeled at the 5′ end with 6-carboxy-fluorescein (6-FAM) and at the 3′ end with 6-carboxy-tetramethyl-rhodamine (TAMRA). Sequences of forward and reverse primers used were, respectively, 5′-TCCTGAAGCAAGTGCATT-3′ and 5′-TATACGCTAAGCCACGTC-3′. A 2 µL template of DNA elution was used for all qPCR amplifications. The amplification program corresponded to a carry-over step of 2 min at 50 °C, followed by an initial denaturation at 95 °C for 10 min and then 40 cycles of 10 s at 95 °C, 30 s at 60 °C and 1 s at 72 °C. Reactions were carried out in triplicate for each sample and each concentration for quantification standard.

#### 2.3.3. Calibration Curves

Calibration curves were built for each type of cheese (FC, SC, SHC) and artificially inoculated with *S. aureus* ATCC29213 reference strain following the method previously described by Kadiroğlu et al. [51]. The pure culture of *S. aureus* ATCC 29213 was obtained by growing strain overnight at 37 °C in brain–heart infusion (BHI) broth, and then serial ten-fold dilutions were performed with physiological water (NaCl 0.9%). Based on the mean values of physico-chemical parameters, one representative standard cheese was selected for each category. In order to obtain stock suspensions (SS) from those, a portion (25 g) of each standard cheese was diluted 10 times in trisodium citrate dihydrate (Na_3_C_6_H_5_O_7_, 2H_2_O) as recommended by the NF EN ISO 8261 standard [52]. SS cheeses were then inoculated with serial dilutions of strain ATCC29213 pure culture to obtain a concentration range from 8 to 2 log CFU/g. The estimated number of CFU was determined by plating 100 µL of the 10^−4^, 10^−5^ and 10^−6^ dilutions on Plate Count Milk Agar (PCMA; Tritium Microbiology B.V., Eindhoven, The Netherlands) after 24 h at 37 °C. A 2 mL aliquot of each concentration from the cheese inoculated suspensions was kept for genomic DNA extraction. The calibration curves for each type of cheese are presented in Figure S1.

### 2.4. Toxigenic Profiles of Isolates

#### 2.4.1. DNA Extraction

Each strain tested was grown on PCMA (AEB 620717, Biomereux Benelux S.A, Bruxelle, Belgium), and DNA was extracted, before performing real-time PCR according to the method developed by the EURL for CPS (https://www.anses.fr/fr/system/files/Liste%20des%20m%C3%A9thodes-LNR%20SCP.pdf, accessed on 12 June 2022) [53].

#### 2.4.2. SEs Production

Isolates were grown in 40 mL of BHI during an incubation at 37 °C for 18 h under rotation at 220 rpm. Cell-free supernatants (CFSs) were then obtained through filtration of the culture broth with 0.22 μm filters (Millex^®^ Gv filter, SLGU033RB). These CFSs were then used for the toxin detection tests described below.

#### 2.4.3. Isolates Characterization

The isolates were characterized using several techniques described below.

##### Rapid Screening Tests

The toxigenic capacity of selected isolates (49) was first rapidly assessed through three routine techniques, advised and used by the EURL for CPS. First, all isolates were screened for the presence of SE-coding genes via a multiplex PCR targeting 11 SE genes (*sea*, *seb*, *sec*, *sed*, *see*, *seg*, *seh*, *sei*, *sej*, *sep* and *ser*) according to the method described by Roussel et al. [30] and for their ability to produce SEs with an immuno-enzymatic test Vidas^®^ SET2 (bioMérieux, Marcy l’Etoile, France) targeting the five classic enterotoxins (SEA, SEB, SEC, SED and SEE). Secondly, isolates that were identified as positive for the presence of at least one of the PCR-targeted genes were subjected to sandwich ELISA tests, available at the EURL for CPS according to the method described by Féraudet et al. [39] in order to confirm expression of and quantify SEA, SEC, SEG and SEI individually.

##### Liquid Chromatography–Mass Spectrometry (LC-MS) Analysis

The CFSs from all 49 isolates were purified and analyzed through liquid chromatography–mass spectrometry (LC-MS), targeting 24 SEs/SEls (SEA, SEB, SEC, SED, SEE, SEG, SEH, SEI, SElJ, SEK, SEL, SEM, SEN, SEO, SEP, SEQ, SER, SES, SET, SElU, SElX, SEY, SElZ and TSST1) according the method developed by the EURL for CPS and reported by Lefebvre et al. [20].

##### Whole Genome Sequencing (WGS)

Genomic DNA was extracted from three to five colonies grown on PCMA (Tritium MicrobiologieB, Ville, The Netherlands) from each of the 49 collected *S. aureus* strains, as detailed above (see Section 2.3.1). DNA integrity was evaluated via electrophoresis on 0.8% agarose gel. DNA libraries were constructed using the Nextera XT DNA Library Prep Kit and sequenced on an Illumina MiSeq (Illumina, San Diego, CA, USA) at the GIGA Genomics platform (Liège, Belgium). Sequencing was performed in paired-end mode at a read length of 300 bp for 15 samples and 150 bp for 34 samples, depending on the fragment length obtained for each genomic DNA isolate after library construction.

Raw reads in *fastq* format were trimmed to remove bases with a Phredscore < 33 and assembled de novo using SPAdes version 3.10.0 [54] with default parameters in the Geneious software version 10.2.3 (https://www.geneious.com, accessed on 5 June 2023) [55]. Annotation of the genomes was performed with Prokka v1.11 [56]. The output Genbank files containing the translations from the annotated coding regions were used as input for NAuRA (Nice automatic Research of alleles) [31]. This tool used BlastP to detect the presence of enterotoxins from a query list of the 33 SEs/SEls and annotated variants against an in-house database of SE protein sequences using a threshold of coverage and identity as described in Merda et al. [31]. Furthermore, to assess the presence of mutated SE genes (e.g., genes carrying a point mutation or gene truncated) in the strains analyzed, BlastN was used. Assembled genomes were aligned against the nucleotide sequence of the 33 SE genes, using 80% as the threshold for coverage and identity in the alignment. The sequence corresponding to the gene was extracted from the genome using bedtools and was inspected for the presence of an open reading frame using the online Expasy translate tool. The results of the analysis for each strain can be found in Table S1.

## 3. Results and Discussion

### 3.1. S. aureus Isolated from the Three Types of Cheese at D0 and DE

#### 3.1.1. Cheeses with Positive Culture at D0 and DE

Among the Belgian cheeses that were included in this study, 10 different varieties were contaminated with CPS (threshold 100 CFU/g). Notably, this represents a lower prevalence than previously observed in artisanal cheeses from raw milk in Brazil, reported in the review by Carneiro Aguiar et al. [13]. The 30 cheese varieties were more contaminated at D0 (*n* = 8) than at DE (*n* = 6), and four varieties were contaminated at both D0 and DE. The identification number (ID) of positive batches for each cheese type for the presence of *S. aureus* at the beginning and the end of shelf life is presented in Table 1. Notably, all of the cheeses with positive cultures were made from either raw cow milk or raw goat milk. Conversely, there was no CPS detected in cheeses that were made from pasteurized milk or ewe milk. 

When possible, one to two colonies (named 1–2) from three samples (named A-C) of each cheese variety were selected. Using this approach, we obtained 34 and 15 isolates from the 19 and 10 contaminated cheese samples at D0 and DE, respectively (Table 1). This difference was principally due to a greater number of positive samples at D0 than at DE.

#### 3.1.2. Quantification of *S. aureus* Strains in Cheeses at D0 and DE

Log CFU/mL in each BP-RPF positive-culture cheese batch at D0 and DE were estimated via quantitative PCR for each cheese matrix (SC, FC and SHC) (Table 1). In all cheese types considered, the levels of *S. aureus* ranged between 3.2 log CFU/mL and 4.7 log CFU/mL. These results have to be interpreted carefully, as the threshold of this method, depending on the different reference matrices, ranged from 3 to 4 log CFU/mL. 

### 3.2. Methods Comparison and Performance

The results obtained showed that isolate 03-A1 D0 tested positive via VIDAS SET2 and negative via PCR for gene *sea* to *see*, but positive for SEP via LC-MS and *sep* via PCR, suggesting a cross-reaction between SEA and SEP (78% to 90% pairwise identities between SEA and SEP [57,58]), as already suggested between SEB and SEG by Hennekinne et al. [59]. In fact, the Vidas SET2 is a rapid and fully automated kit using a cone coated with a mixture of antibodies specific for SEA, SEB, SECs, SED and SEE. This immunological tool has a great sensitivity for detection of SEA to SEE in food [59,60]. However, mismatches between PCR and the VIDAS SET2 test can happen and could be explained by the cross-reaction with other SEs that exhibit a high degree of homology or by variant *se* genes not recognized by the primers used [59]. 

For the classic toxins detected in our study (SEA and SEC), the information obtained through immunological methods such as VIDAS SET2 and ELISA agreed with the molecular assays and chemical method LC-MS, with the exception of strain 27A-1 D0. Although 27A-1 D0 is characterized by the *sec15* allele, SEC was not detected, neither through VIDAS SET2, nor through ELISA, nor through LC-MS. This may be explained by the absence or low expression of this specific allelic gene. 

LC-MS (threshold > 1 ng/mL) was not always able to detect SEG and SEI in strains harboring *seg* and *sei*, while ELISA (threshold > 0.01 ng/mL) does. This can be explained by the lack of sensitivity of LC-MS and the production of SEG and SEI at a concentration below the LOD of LC-MS (1 ng/mL) [32]. Moreover, LC-MS was not able to detect SEI from the *sei8* allele, despite ELISA results indicating that it was present at a maximum concentration of 1.4 ng/mL. 

In 16.7% of the varieties of Belgian cheese made from raw milk, we found strains possessing an *seg* truncated gene (*seg*T) (Table 2) with a predicted protein of 191 amino acids (SEGT), which exhibited an SEG that was truncated in the C terminal relative to a non-truncated SEG. Strains with similar gene profiles were previously classified by their *vSaβ* type and characterized as being low or high producers of SEI and SEG [44]. SEGT was not detected via ELISA, but LC-MS detected SEGT in 33.3% of strains possessing *seg*T (Table 2). The *seg*T does not possess the sequence of the domains needed for superantigenic action, but there is currently no evidence that emetic action is lost due to the truncation. It should be noted that one of the features of emesis induction may be a disulfide bond between two cysteine residues, but the exact mechanism of emetic activity still remains unclear [1,61]. The bond between two cysteine residues can theoretically still be present in SEGT. Furthermore, it has been shown that denatured toxins in serologically negative samples still retain enough biological activity to cause an illness [62]. Furthermore, Ono et al. [61] showed that there was no complete relationship between emetic and superantigenic activities. The existence of *seg*T may complicate the interpretation of the results on the prevalence and expression of the *seg* gene, because the strains harboring *seg*T were positive to multiplex real-time PCR to *seg*, and ELISA was unable to detect SEGT.

### 3.3. Toxin Production and SE Gene Profiles in Belgian Cheese Varieties

Looking only at cheese made from raw milk (*n* = 24), only 8.3% of the varieties with *S. aureus* carried classic SEs, and 29.2% carried *egc*-encoded SEs (Table 3). 

Only two classic enterotoxin genes, *sea* and *sec*, were detected and were at a low prevalence, with only 4 and 18.3% of the strains, respectively, corresponding to only one and two cheeses (made from raw cow and goat milk), respectively. With one exception, these were always associated with the detection of the toxin (Table 3). As previously described in other studies, *sel* and *sec* occur together [63], and SEL was always detected when *sel* was present. 

The *egc*-encoded SE genes (*seg*, *sei*, *sem*, *sen*, *seo* or *selu*) were present in at least 55.1% of the *S. aureus* strains isolated from Belgian artisanal cheeses, which is consistent with the existing literature [4,44]. In other words, 7 out of 10 cheese varieties that were positive for CPS carried *egc-en*coded SEs, and these were all made from raw cow milk. These genes were present together with three distinct association patterns: (i) *seg*T, *sei*, *sen*, *seo*, *selu*; (ii) *seg*T, *sei*, *sem*, *sen*, *seo*, *selu* and (iii*) seg*, *sei*, *sem*, *sen*, *seo*, *selu*, with varying toxin detection rates (100% to 3.7%) in strains carrying the egc-encoded genes (Table 2). This is consistent with the fact that the structure of the *egc* locus is variable [64], and to our knowledge, these particular profiles have not yet been described. In our study, SEI and SEG were detected (through ELISA) in 100% and 33.3% of strains harboring *sei* and *seg*, respectively. This observation is partially in agreement with Omoe et al. [65], who showed that less than 60% of the *S. aureus* isolates harboring *sei* and *seg* produced a detectable level of SEG or SEI. For the four other *egc*-encoded SE genes (*sem*, *sen*, *seo* and *selu*) detected in strains, the SEs were only detected via the LC-MS method and were detected only in a few cases (Table 2). 

The *selx* gene was present in 100% of the strains studied, which is in agreement with the prevalence presented by Fisher et al. [1], with toxin detection occurring in 81.6% of cases (Table 2). For the *sey* gene, the prevalence among strains was not consistent with Fisher et al. [1]. In our study, the *sey* gene was present in 48.9% of the strains, corresponding to four different varieties of cheese that were positive for CPS, while Fisher et al. [1] only reported it in a minority of isolates. This observation is important, because SEY has a demonstrated emetic effect [42]. The detection of *tsst1* was always associated with toxin detection and was detected in two varieties of cheese, while it was never detected in the study by Chieffi et al. [66] in raw milk samples. 

In our study, eight different toxin gene profiles were identified among the 49 *S. aureus* strains tested (Table 3). Other studies also described the diversity of strains of *S. aureus* in dairy products and showed that that there was a wide variety of genetic profiles found in dairy products’ CPS under natural conditions. Non-classic SE and SEl genes were the most prevalent. The association *sed*, *selj*, *ser* has regularly been found in strains from dairy products and raw milk in other studies [45,66], but none of the strains in this study exhibited this profile. However, given the diversity of genotyping techniques available [45,66,67,68] and the choice of genes analyzed in different studies, a robust comparison of SE genetic profiles is difficult at this stage.

## 4. Conclusions

According to the results of this study, the classic toxins SEA to SEE are not the dominant toxins in the Belgian artisanal cheeses analyzed in this study, while all strains harbored at least one SE gene from the group of so-called “new SE and SE*l*s”. Notably, some of them have shown emetic activity, and there is evidence of their involvement in SFP outbreaks. However, currently, there is a lack of tools to facilitate the direct detection of these new enterotoxins in food, as commercially available kits are only available for the classic enterotoxins (SEA to SEE). Therefore, it is important to carry out specific studies on the emetic activities of these new enterotoxins and to develop rapid screening methods to detect them directly in food matrices. Indeed, the lack of routine detection methods for these toxins may lead to a failure to detect SE(l)s in food and increase the risk of FPOs with an undefined etiology. 

Following this study, it would be interesting to investigate the parameters favoring the production of these toxins in cheese made from raw milk during the first stages of production and ripening. For that, different strains producing new SEs and SE*l*s could be used: (1) strains naturally present in cheeses and (2) strains isolated during SFP outbreaks associated with cheeses and dairy products.

## Figures and Tables

**Table 1:** Varieties of cheeses positive for the presence of CPS at D0 and DE, number of strains isolated and CPS concentration for each contaminated variety in CFU/mL.

Variety n°	Type of Milk	Origin of Milk	Type of Cheese	End of Production (D0)		Expiry Date (DE)	
				Log CFU/mL in SS at D0	Number of Positives Samples and Number of Isolates *	Log CFU/mL in SS at DE	Number of Positives Samples and Number of Isolates *
1	R	cow	SC				
2	R	cow	FC				
3	R	cow	FC	4	3 (2, 2, 2)	4.2	1 (1, 0, 0)
4	P	cow	FC				
5	P	ewe	FC				
6	R	goat	SC				
7	R	cow	FC				
8	R	cow	FC				
9	P	ewe	SHC				
10	R	ewe	SHC				
11	R	cow	SHC				
12	R	cow	SHC				
13	R	goat	FC	3.9	1 (1, 0, 0)	4.7	2 (1, 0, 1)
14	P	cow	SHC				
15	P	cow	SHC				
16	R	goat	FC				
17	R	cow	FC				
18	R	cow	SHC	ND	1 (0, 0, 1)		
19	P	cow	SC				
20	R	cow	SC				
21	R	cow	SHC				
23	R	cow	SHC	4	3 (2, 2, 1)	4	3 (2, 2, 2)
24	R	cow	SHC			3.5	1 (0, 1, 0)
25	R	cow	FC				
26	R	cow	SHC			3.2	1 (0, 0, 1)
27	R	cow	SC	4.4	3 (2, 2, 2)		
28	R	cow	FC	3.6	2 (1, 2, 0)		
29	R	cow	SC	3.7	3 (2, 2, 2)	4.3	2 (2, 0, 2)
31	R	cow	SC				
32	R	cow	SHC	4.8	3 (2, 2, 2)		
TOTAL	positive	samples	(isolates)		19 (34)		10 (15)

* Number of positives samples and number of isolates: The number of isolates obtained from each sample (A, B and C) is indicated, presented as X(x_1_, x_2_, x_3_). X: number of cheese samples (i.e., replicates) contaminated with CPS. x_1_: number of isolates from replicate A. x_2_: number of isolates from replicate B. x_3_: number of isolates from replicate C. FC: fresh cheese. SC: soft cheese. SHC: semi-hard cheese. R = raw. P = pasteurized. ND: *S. aureus* not detected via qPCR. The qPCR was not performed in samples where no CPS colony had grown.

**Table 2:** Frequency of profiles of *SE* genes detected using NAuRA genomic tool and their associated toxins detected via LC-MS in the 49 strains collected in 3 different types of raw milk cheeses among the 24 cheese varieties in Belgium.

Type of Cheese	Type of Milk	Profiles of *SE* Genes (NaURA)	Frequency of *SE* Genes in Strains Characterized	Frequency of *SE* Genes Profiles in Cheeses Varieties (ID)	Toxins Produced by the Strain in BHI Culture (LC-MS)
FC	Goat	*sea*, *sec*, *sel*, *sex*, *sew*, *tsst1*	2/49	1/24 (13)	SEA, SEC, SEL, TSST1
FC	Goat	*sec*, *sel*, *sex*, *sew*, *tsst1*	1/49	1/24 (13)	SEC, SEL, SElX, TSST1
FC-SC	Cow	*selx*	19/49	3/24 (03, 28, 29)	SElX
SHC	Cow	*seg*T, *sei*, *sen*, *seo*, *selu*, *sex*, *sey*, *sez*	6/49	1/24 (32)	SEG, SEI, SElX, SEY, SElZ SEG, SEY, SElZ
FC	Cow	*seg*, *sei*, *sem*, *sen*, *seo*, *sep*, *selu*, *sex*	1/49	1/24 (03)	SEG, SEI, SEM, SEN, SEO, SEP, SElX
SHC	Cow	*seg*, *sei*, *sem*, *sen*, *seo*, *selu*, *sex*, *sew*	2/49	2/24 (18, 26)	SEI, SEM, SElU, SElX SEI, SEM, SEO, SElU, SElX
SC	Cow	*sec*, *seg*T, *sei*, *sel*, *sen*, *seo*, *selu*, *sex*, *sey*, *sez*, *tsst1*	6/49	1/24 (27)	SEL, SEY, SEZ, TSST1 SEC, SEI, SEL, SEX, SEY, SEZ, TSST1 SEC, SEL, SEX, SEY, SEZ, TSST1
SHC	Cow	*seg*T, *sei*, *sem*, *sen*, *seo*, *selu*, *sex*, *sey*, *sez*	12/49	2/24 (23, 24)	SEG, SEM, SEO, SEY, SEZ SEG, SEI, SEX, SEY, SEZ SEG, SEY, SEZ SEX, SEY, SEZ SEI, SEX, SEY, SEZ SEY, SEZ

Legend: FC: fresh cheese; SC: soft cheese; SHC: semi-hard cheese; ID: identification number of cheese varieties. SEW toxin was not studied via LC-MS.

**Table 3:** Summary of the frequencies of the different SE genes and their corresponding toxins in the different varieties of raw milk cheeses (*n* = 24) at D0 and DE.

SE Genes	*sea*	*sec*	*seg*	*seg*T	*sei*	*sel*	*sem*	*sen*	*seo*	*sep*	*selu*	*sew*	*selx*	*sey*	*selz*	*tsst1*
Number of variety of Belgian cheese containing the SE gene	1	2	3	4	7	2	5	7	7	1	6	3	10	4	4	2
% raw milk cheese containing the SE gene ^(a)^ (*n* = 24)	4.1	8.3	12.5	16.7	29.2	8.3	20.8	29.2	29.2	4.1	25	12.5	41.7	16.7	16.7	8.3
Number of strains containing the SE gene (*n* = 49)	2	9	3	24	27	9	15	27	27	1	26	5	49	24	24	9
Occurrence of toxin detection via LC-MS and via ELISA when gene toxin is present (%)	100 ^(b)^ 100 ^(c)^	88.9 ^(b)^ 88.9 ^(c)^	33.3 ^(b)^	33.3 ^(b)^	55.5 ^(b)^ 100 ^(c)^	100 ^(b)^	26.7 ^(b)^	3.7 ^(b)^	11.1 ^(b)^	100 ^(b)^	7.7 ^(b)^	NA	81.6 ^(b)^	100 ^(b)^	100 ^(b)^	100 ^(b)^

^(a)^ the 6/30 cheeses made from pasteurized milk were not included, because they were all negative for SE genes. ^(b)^ via LC-MS. *^(^*^c)^ via ELISA. NA: Not applicable, the toxin is not being investigated^.^

## Data Availability

The data presented in this study are available on request from the corresponding author.

## Supplementary Materials

The following supporting information can be downloaded at: https://www.mdpi.com/article/10.3390/foods12214019/s1, Figure S1: Standard curve for nuc gene qPCR of cheese suspensions inoculated with S. aureus ATCC29213; Table S1: Identification of genes coding for enterotoxins with the NAuRA genetic tool in 49 strains of the *S. aureus* isolated from the different Belgian artisanal cheeses. Each number corresponds to an allele variant coding for an enterotoxin. When toxin was detected via LC-MS, the letter T was added. NA: non-applicable, toxin not searched. +: SEG truncated gene; Table S2: Description of the main characteristics of the different cheese varieties studied.
