# Supplementary material for: The Enterotoxin Gene Profiles and Enterotoxin Production of Staphylococcus aureus Strains Isolated from Artisanal Cheeses in Belgium
Source: Foods. 2023 Nov 3;12(21):4019. doi: 10.3390/foods12214019 (PMC10650413; doi:10.3390/foods12214019)
Supplement: Supplementary file 1 [file foods-12-04019-s001.zip › foods-2669639-supplementary.pdf]

foods

MDPI

Supplementary Material

The Enterotoxin Gene Profiles and Enterotoxin Production of *Staphylococcus aureus* Strains Isolated from Artisanal Cheeses in Belgium

Raphaëlle Minutillo [1†], Barbara Pirard [1†], Abdelhak Fatihi [2], Marina Cavaiuolo [2], Donatien Lefebvre [2], Amaury Gérard [3], Bernard Taminiau [1], Yacine Nia [2], Jacques-Antoine Hennekinne [2], Georges Daube [1] and Antoine Clinquart [1*]

[1] Department of Food Sciences, Fundamental and Applied Research for Animals & Health (FARAH), Faculty of Veterinary Medecine, Avenue de Cureghem 10, 4000 Liege, Belgium; babs.pirard@gmail.com (B.P.); bernard.taminiau@uliege.be (B.T.); georges.daube@uliege.be (G.D.)
[2] Laboratory for Food Safety, French Agency for Food, Environmental and Occupational Health & Safety (ANSES), Université Paris-Est, 94700 Maisons-Alfort, France; abdelhak.fatihi@anses.fr (A.F.); marina.cavaiuolo@anses.fr (M.C.); donatien.lefebvre@catalent.com (D.L.); yacine.nia@anses.fr (Y.N.); jacques-antoine.hennekinne@anses.fr (J.-A.H.)
[3] Brewing and Food Science Unit, LABIRIS, Avenue Emile Gryzon 1, 1070 Anderlecht, Belgium; agerard@spfb.brussels
* Correspondence: antoine.clinquart@uliege.be
† These authors contributed equally to this work.

Table S1. Identification of genes coding for enterotoxins with the NAuRA genetic tool in 49 strains of the *S. aureus* isolated from the different Belgian artisanal cheeses. Each number corresponds to an allele variant coding for an enterotoxin. When toxin was detected via LC-MS, the letter T was added. NA: non-applicable, toxin not searched. +: SEG truncated gene.

Strain	SEA	SEC	SEG	SEI	SEL	SEM	SEN	SEO	SEP	SElU	SElX	SEY	SElW	SElZ	TSST1
03A-1 D0			3/T	4/T		23/T	3/T	3/T	2/T		9/T				
03A-1 DE											6/T				
03A-2 D0											6/T				
03B-1 D0											6/T				
03B-2 D0											6/T				
03C-1 D0											6/T				
03C-2 D0											6/T				
13A-1 DE		14/T			2/T						7/T		13/NA		2/T
13B-1 D0	2/T	2/T			2/T						7		13/NA		2/T
13C-1 DE	2/T	2/T			2/T						7		13/NA		2/T
18C-1 D0			16	14/T		22/T	15	11		12/T	31/T		28/NA		
23A-1 D0			+/T	8/T		21	6	5		9	12/T	4/T		1/T	
23A-1 DE			+	8		21	6	5		9	12/T	4/T		1/T	
23A-2 D0			+	8/T		21	6	5		9	12/T	4/T		1/T	
23A-2 DE			+	8		21	6	5		9	12/T	4/T		1/T	
23B-1 D0			+	8/T		21	6	5		9	12/T	4/T		1/T	
23B-1 DE			+	8		21	6	5		9	12	4/T		1/T	
23B-2 D0			+	8		21	6	5		9	12/T	4/T		1/T	
23B-2 DE			+	8		21	6	5		9	12	4/T		1/T	
23C-1 D0			+	8/T		21	6	5		9	12/T	4/T		1/T	
23C-1 DE			+	8		21	6	5		9	12/T	4/T		1/T	

23C-2 DE		+/T	8		21	6	5		9	12	4/T		1/T	
24B-1 DE		+/T	8		21/T	6	5/T		9	12	4/T		1/T	
26C-1 DE		16	14/T		22/T	15	11/T		12/T	31/T		28/NA		
27A-1 D0	15	+	8	1/T		6	5		9	12	4/T		1/T	5/T
27A-2 D0	7/T	+	8/T	1/T		6	5		9	12/T	4/T		1/T	5/T
27B-1 D0	7/T	+	8/T	1/T		6	5		9	12/T	4/T		1/T	5/T
27B-2 D0	7/T	+	8	1/T		6	5		9	12/T	4/T		1/T	5/T
27C-1 D0	7/T	+	8/T	1/T		6	5		9	12/T	4/T		1/T	5/T
27C-2 D0	7/T	+	8	1/T		6	5		9	12	4/T		1/T	5/T
28A-1 D0										56/T				
28B-1 D0										56/T				
28B-2 D0										56/T				
29A-1 D0										6/T				
29A-1 DE (low coverage)										6/T				
29A-2 D0										6/T				
29A-2 DE (low coverage)										6/T				
29B-1 D0 (contamination)										6/T				
29B-2 D0										6/T				
29C-1 D0										6/T				
29C-1 DE										6/T				
29C-2 D0										6/T				
29C-2 DE										6/T				
32A-1 D0		+/T	8			6	27		9	12	4/T		1/T	
32A-2 D0		+/T	8/T			6	5		9	12/T	4/T		1/T	
32B-1 D0		+/T	8/T			6	5		9	12/T	4/T		1/T	
32B-2 D0		+/T	8/T			6	5		9	12/T	4/T		1/T	
32C-1 D0		+/T	8/T			6	5		9	12/T	4/T		1/T	
32C-2 D0		+/T	8/T			6	5		9	12/T	4/T		1/T	

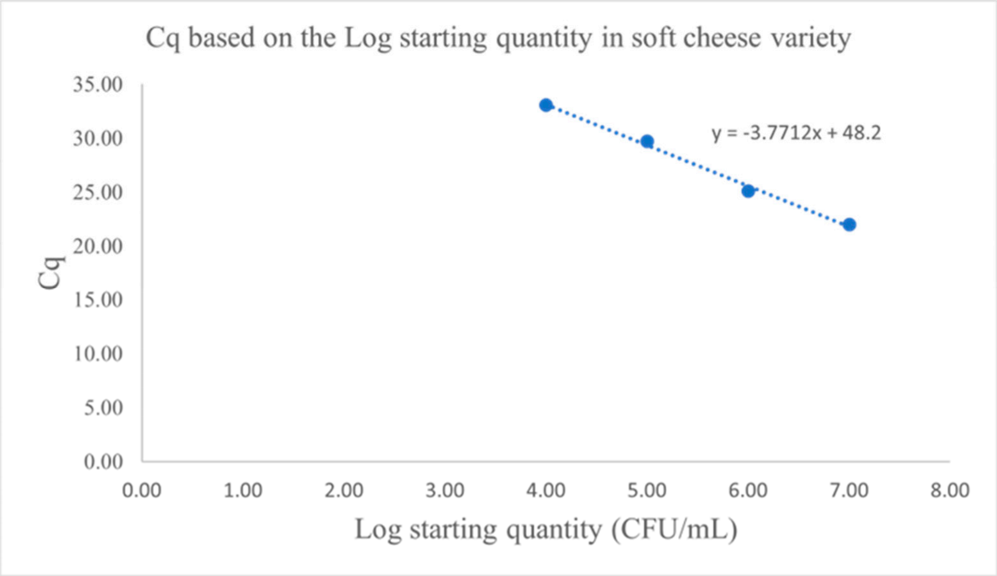
Cq based on the Log starting quantity in soft cheese variety
35.00
30.00
25.00
20.00
15.00
10.00
5.00
0.00
Cq
y = -3.7712x + 48.2
0.00
1.00
2.00
3.00
4.00
5.00
6.00
7.00
8.00
Log starting quantity (CFU/mL)

a

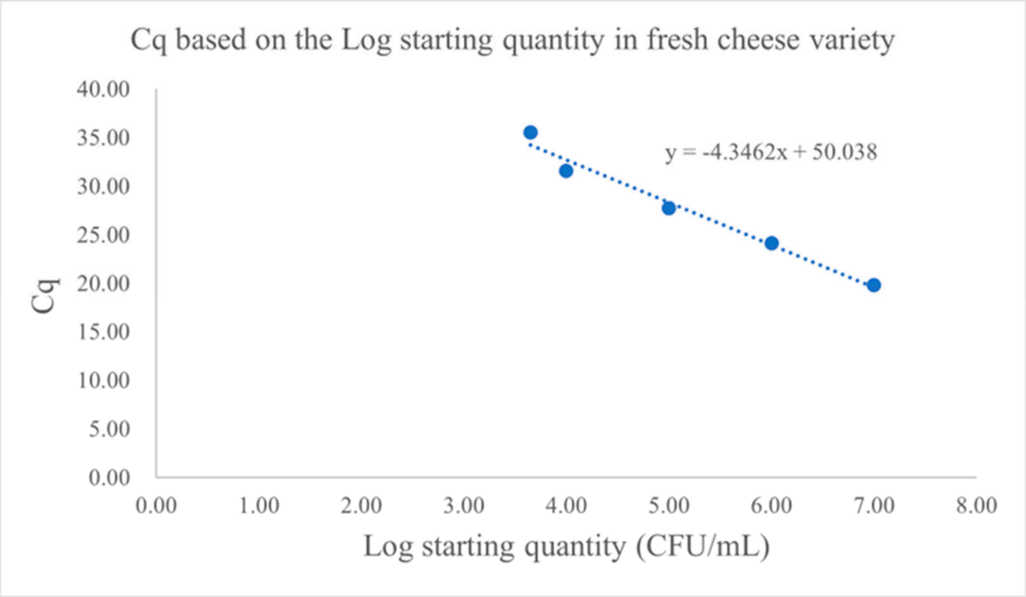
Cq based on the Log starting quantity in fresh cheese variety
40.00
35.00
30.00
25.00
20.00
15.00
10.00
5.00
0.00
Cq
y = -4.3462x + 50.038
0.00
1.00
2.00
3.00
4.00
5.00
6.00
7.00
8.00
Log starting quantity (CFU/mL)

b

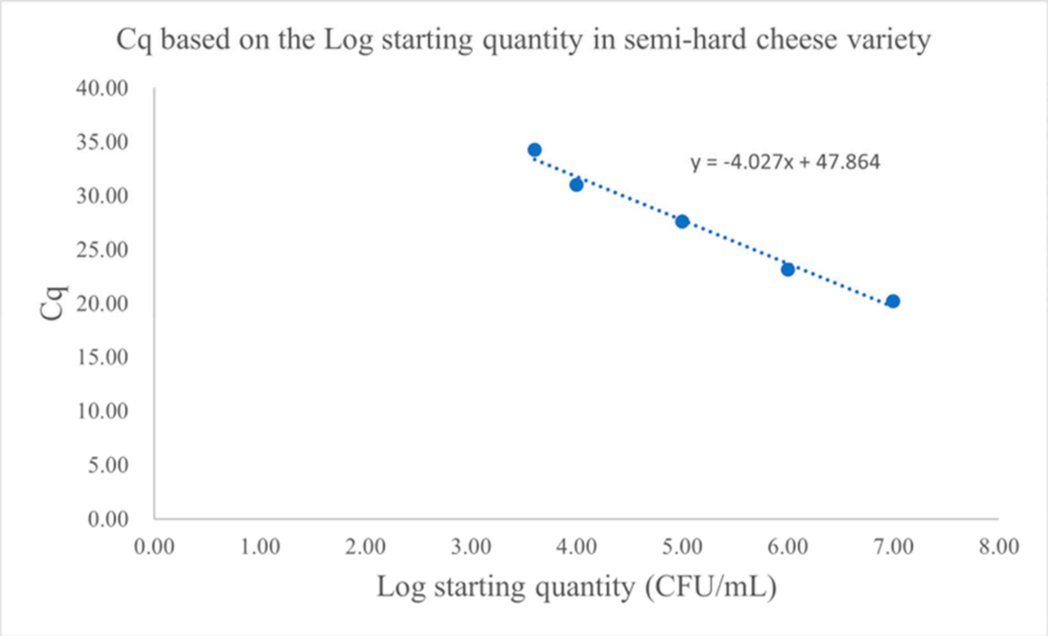

c

Figure S1. Standard curve for *nuc* gene qPCR of cheese suspensions inoculated with *S. aureus* ATCC29213.

Table S2. Description of the main characteristics of the different cheese varieties studied.

Variety	Type of cheese	Sub-type of cheese	Ripening time (weeks)	Shelf life (days)	Origin of milk	Type of milk
1	SC	Smear-coat	3	40	Cow	Raw
2	FC	Type maquée	0	19	Cow	Raw
3	FC	Type maquée	0	14	Cow	Raw
4	FC	Type maquée	0	16	Cow	Pasteurized
5	FC	Mold-drained	0	10	Ewe	Pasteurized
6	SC	Bloomy rind	2	30	Goat	Raw
7	FC	Type maquée	0	7	Cow	Raw
8	FC	Type maquée	0	12	Cow	Raw
9	SHC	Type Gouda	8	21	Ewe	Pasteurized
10	SHC	Type Saint-Paulin	12	21	Ewe	Raw
11	SHC	Type Saint-Paulin	6	14	C	Raw
12	SHC	Curd-pressed	24	14	Cow	Raw
13	FC	Fresh	0	15	Goat	Raw
14	SHC	Type Gouda	8	21	Cow	Pasteurized
15	SHC	Type Gouda	3	30	Cow	Pasteurized
16	FC	Mold-drained	0	8	Goat	Raw
17	FC	Mold-drained	0	14	Cow	Raw
18	SHC	Type Gouda	6	21	Cow	Raw
19	SC	Bloomy rind	3	30	Cow	Pasteurized
20	SC	Bloomy rind	2	30	Cow	Raw
21	SHC	Type Saint-Paulin	3	21	Cow	Raw
22	FC	Type maquée	0	10	Goat	Raw
23	SHC	Type Saint-Paulin	6	30	Cow	Raw

24	SHC	Type Saint-Paulin	2	21	Cow	Raw
25	FC	Type maquée	0	10	Cow	Raw
26	SHC	Type Saint-Paulin	5	14	Cow	Raw
27	SC	Smear-coat	2	30	Cow	Raw
28	FC	Type maquée	0	10	Cow	Raw
29	SC	Bloomy rind	3	28	Cow	Raw
30	SC	Smear-coat	4	30	Cow	Raw
31	SC	Smear-coat	3	40	Cow	Raw
32	SHC	Type Saint-Paulin	3	14	Cow	Raw

Legend: SC: soft cheese; FC: fresh cheese; SHC: semi-hard cheese. In Belgium, 'type maquée' is a typical white cheese/cottage cheese originating from Wallonia. Lost samples are shown in grey.
